# Suicide by pesticide ingestion in Nepal and the impact of pesticide regulation

**DOI:** 10.1186/s12889-021-11155-3

**Published:** 2021-06-14

**Authors:** Leah Utyasheva, Dilli Sharma, Rakesh Ghimire, Ayanthi Karunarathne, Gael Robertson, Michael Eddleston

**Affiliations:** 1 Centre for Pesticide Suicide Prevention, University of Edinburgh, QMRI E3.22a, 47 Little France Crescent, Edinburgh, EH16 4TJ UK; 2 Maharajgunj Medical Campus, Institute of Medicine, Tribhuvan University, Kathmandu, Nepal; 3 Pharmacology, Toxicology & Therapeutics, University/BHF Centre for Cardiovascular Science, University of Edinburgh, Edinburgh, UK

**Keywords:** Nepal, Suicide, Poisoning, Pesticides, Means restriction

## Abstract

**Background:**

Nepal recorded 5754 suicides in 2018–19 - a high number for a relatively small country. Over 24% of these suicides were by poisoning, most by ingestion of highly concentrated agricultural pesticides. Nepal has actively regulated pesticides to reduce their health impacts since 2001. We aimed to analyse Nepal’s history of pesticide regulation, pesticides responsible for poisonings, and relate them to national suicide rates.

**Methods:**

Information on pesticide regulation was collected from the Plant Quarantine and Pesticide Management Centre of the Ministry of Agriculture and Livestock Development. National data on suicides from 1980 to 2019 were obtained from the National Statistical Bureau and Nepal Police. Data on the pesticides responsible for self-poisoning and pesticide suicides over time were obtained from a systematic literature review.

**Results:**

As of June 2020, 171 pesticides were registered for use in Nepal, of which one was extremely hazardous (WHO Class Ia), one other highly hazardous (WHO Class Ib), and 71 moderately hazardous (WHO Class II). Twenty-four pesticides have been banned since 2001, with eight (including five WHO Class I compounds) banned in 2019. Although the suicide rate has increased more than twelve-fold since 1980, particularly for hanging (15-fold increase from 1980 to 2018), fatal pesticide self-poisoning has increased by 13-fold. Methyl-parathion is reported to be the key pesticide responsible for pesticide self-poisoning in Nepal, despite being banned in 2006.

**Conclusion:**

The full effect of the recent pesticide policy reform in Nepal remains to be seen. Our analysis shows a continuing increase in suicide numbers, despite bans of the most important pesticide in 2006. This may indicate smuggling across the border and the use of the brand name (Metacid) for pesticides in general making it difficult to identify the responsible pesticide. More information is required from forensic toxicology labs that identify the individual compounds found. The effect of recent bans of common suicide pesticides needs to be monitored over the coming years. Evidence from other Asian countries suggests that HHPs bans will lead to a marked reduction in suicides, as well as fewer cases of occupational poisoning.

## Introduction

Pesticide poisoning affects peoples’ lives and health, particularly in low- and middle-income countries (LMIC) where high proportions of the population are engaged in agriculture and use highly hazardous pesticides (HHPs [1, 2]) on a daily basis. There is a growing international acceptance that rapidly reducing and progressively eliminating exposure to toxic chemicals is essential for the protection of human rights, health, and lives, and for achieving the Sustainable Development Goals (SDGs) [3, 4]. Both the World Health Organization (WHO) and Food and Agriculture Organization of the United Nations (FAO) stress their commitment to pesticide risk reduction, including a progressive ban of HHPs [5].

HHPs have been responsible for an estimated 14 million premature deaths from pesticide suicide since the Green Revolution placed them into rural households completely unable to use or store them safely [6]. Data from Sri Lanka [7–9], Bangladesh [10], South Korea [11] and Kerala (India) [12] indicate that pesticide regulation to remove HHPs from agriculture results in marked reductions in pesticide suicides without apparent effect on agricultural yield. Means restriction works for highly lethal suicide methods, such as poisoning with HHPs, because it puts space and time between the person and means, allowing the suicide impulse to pass or selection of a less lethal means, increasing the chance of survival [13, 14].

Nepal is a South Asian LMIC with the population of 29 million that is heavily dependent on agriculture. According to the WHO, it has a high suicide rate of around 20/100,000 per year in 2012 [13]. Poisoning is a common cause of suicide [15–17], with pesticides the most important poison [18, 19]. Pesticides are commonly used in Nepalese agriculture; they are regulated by the Plant Quarantine and Pesticide Management Centre (PQPMC) within the Ministry of Agriculture and Livestock Development (Box 1).

There are no nationwide data on the incidence of acute pesticide poisoning or of the pesticides causing deaths in Nepal [16, 20]. The Ministry of Health and Population collects data on poisoning but groups all poisons and forms (intentional, accidental, or occupational) of poisoning together, limiting the possibility for analysis. The Nepal Police records the numbers of suicides by poisoning, but again does not distinguish the agent. The Government of Nepal’s Heath Management Information System (HMIS) does not include the detailed information on poisoning and suicides that would make comprehensive data collection possible. Record-keeping at hospitals is under-resourced, limiting its usefulness for analysis of the precise poisons involved in cases and deaths [20].

The aim of this work was to identify the pesticide regulation that has been performed in Nepal, to identify pesticides responsible for most poisonings, and to relate these to the incidence of poisoning suicides over the last 40 years in the country.

### Text Box 1. Pesticide regulation in Nepal

Nepal passed its first Pesticide Act in 1991 [21]. The Pesticide Rules were approved in January 1993 and became operative with the Act on 16 July 1994. The Act regulates the import, manufacture, sales, distribution and use of pesticides within Nepal. It also established a Pesticide Committee composed of members from various ministries, the Pesticide Association of Nepal, scientists and consumer groups for the purpose of managing pesticide-related issues.

In 2015–2019, Nepal underwent an administrative and government reform, adopting a new Constitution in 2015, new Criminal and Civil Codes in 2017, and importantly a new Pesticide Management Act in 2019. The new Constitution changed the unitary administrative system to a federal system, with governance at federal, provincial, and municipal levels.

Pesticide management is delegated to the PQPMC of the Ministry of Agriculture and Livestock Development (MoALD). It was set up as part of structural reforms in 2018 to strengthen the government’s commitment to pesticide management. It is responsible for federal pesticide management to oversee the implementation of the Pesticide Management Act including registration, re-registration, de-registration, and banning of pesticides as well as the coordination and administration of the Pesticide Management Committee. PQPMC is Nepal’s Designated National Authority for the Rotterdam Convention.

Nepal’s provinces obtained a mandate to engage with pesticide management with the new legislation, with creation of provincial Ministries for Land Management, Agriculture and Cooperative. Provinces can formulate their own Pesticide Committees and have powers to renew pesticide licenses. There are two bodies under each provincial Ministry responsible for pesticide management – Provincial Training Directorate and Provincial Agriculture Development Directorate (PADD). Each PADD has a number of Agriculture Knowledge Centres (AKCs) established in place of previous District Agriculture Development Offices. The AKCs are involved in knowledge sharing related to production of agricultural products, and in connecting researchers and farmers, and transferring technology by training.

According to the law, provincial powers include re-registration of pesticides (as opposed to registration, which is possible only on the federal level), registration of home-made botanical pesticides and promotion of bio-pesticide production in the country, as well as strengthening provisions for pesticide disposal. Provincial pesticide management committees can also issue licenses for distribution, storing, use and application of pesticides. The new provincial mandate is still under development.

In 2016, Nepal also developed a voluntary Code of Practice for Using Pesticides – a non-binding document based on the FAO’s International Code of Conduct on Pesticide Management [22]. The Code of Practice provides practical guidance to public and private sector organisations involved in the pesticide life-cycle, from production (manufacture and formulation) to disposal. It aims to minimize adverse health and environmental effects as well as human and animal exposure. It is intended to serve as a guiding framework for strengthening the capacity of stakeholders to regulate, evaluate, and enforce effective control over pesticides.

## Methods

Nepal’s data on pesticide registration, use, and bans were obtained from the PQPMC website and from PQPMC officials. Suicide data were extracted from the Police and National Statistical Bureau reports for the years 1980 to 2019. The methods used in recording suicides are classified into seven categories: hanging, poisoning, weapon and instruments, drowning, burning, jumping, and electric current. The poisoning classification includes poisoning with substances other than pesticides (i.e. drugs and medicines). We used the World Bank data on the population of Nepal for each year from 1980 to 2019 as the denominator to calculate crude suicide rates.

We reviewed the literature for all papers reporting pesticide poisoning in Nepal from 1980 to 2019. We hand-searched Nepalese language and national journals for studies. We systematically searched PubMed and www.google.com for studies on pesticide and poisoning in Nepal using the search terms “Nepal” and “poisoning”. We selected English language papers reporting primary studies with the aim to identify the compounds responsible for pesticide poisoning. There were no publication date restrictions. We reviewed the first 150 hits found on www.google.com until no new studies were revealed compared to the PubMed search.

### Statistical analysis

Simple descriptive statistics were used to describe the data.

## Results

### Pesticides

**Fig. 1:**
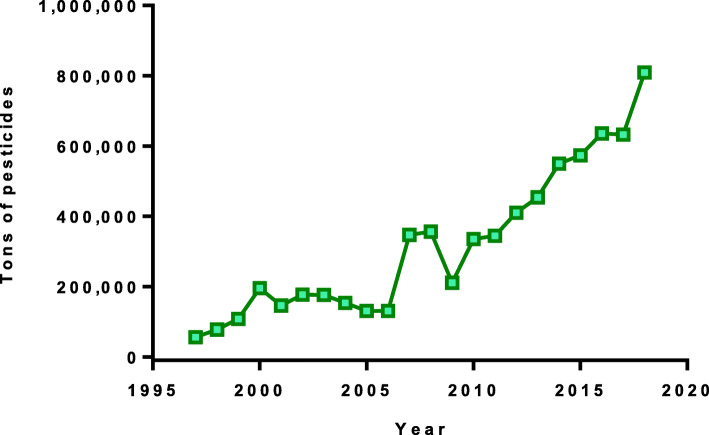
National trends of pesticide imports. (Source: Plant Quarantine and Pesticide Management Centre, data available from authors upon request)

Nepal uses relatively little pesticide compared to other countries. According to the Ministry of Agriculture and Livestock Development, the country uses 0.396 active ingredient (a.i.) kg of pesticide per hectare of land, in comparison to 0.481 a.i. kg/ha in India and 1.9 kg/ha in Europe [23]. However, pesticide imports are increasing (from 56 metric tons in 1997–8 to 809 tons in 2018–19, Fig. 1). Gross sales and values account for US $7.5 million per year. These values do not take into account smuggling of pesticides across the border from India [21].

There are currently 171 pesticides registered in the country. According to the WHO hazard classification [24], one (0.6%) is extremely hazardous (WHO class Ia), one (0.6%) highly hazardous (WHO class Ib), 73 (42.44%) moderately hazardous (WHO class II), 28 (16.27%) slightly hazardous (WHO class III), and 68 unlikely to present acute hazard (WHO class U) (Table 1).

**Table 1:** Classification of registered pesticides by WHO toxicity category

WHO class	Insecticide	Acaricide	Fungicide	Herbicide	Rodenticide	Biopesticide	Others^a^	Total
**Ia**					1			1
**Ib**					1			1
**II**	43	3	15	11			1	73
**III**	11	1	6	7		1	2	28
**NC**	1							2
**U**	4	2	22	12		13	13	66
**Total**	61	6	43	30	2	14	16	171

^a^ **Others** includes bactericides, herbal pesticides, molluscicides, and nematicides

WHO hazard class Ia pesticide: 1; class Ib pesticides: the anticoagulant rodenticide bromadiolone (rodenticide) is a hazard class Ia compound while the rodenticide zinc phosphide is hazard class Ib.

Twenty-four pesticides have been banned in Nepal, the majority are persistent organic pollutant (POP) organochlorine compounds that were banned in 2001 and 2019 following adoption of the Stockholm Convention on Persistent Organic Pollutants (Table 2). Ten of the banned pesticides are obsolete and not used in agriculture and public health. In 2006, methyl parathion and monocrotophos were banned; two other acutely toxic HHPs (endosulfan and phorate) were banned in 2012 and 2015.

**Table 2:** Pesticides banned in Nepal

Pesticide	Year	WHO hazard class	Cited reason for ban
Chlordane	2001	II	Complying with Stockholm Convention banning of Persistent Organic Pollutant
Dieldrin	2001	(O) Obsolete not classified	Complying with Stockholm Convention banning of Persistent Organic Pollutant
Aldrin	2001	(O) Obsolete not classified	Complying with Stockholm Convention banning of Persistent Organic Pollutant
Mirex	2001	(O) Obsolete not classified	Complying with Stockholm convention banning of Persistent Organic Pollutant
Lindane	2001	II	Complying with Stockholm convention banning of Persistent Organic Pollutant
Phosphamidon	2001	Ia	Complying with Stockholm convention banning of Persistent Organic Pollutant
DDT	2001	II	Complying with Stockholm convention banning of Persistent Organic Pollutant
Endrin	2001	(O) Obsolete not classified	Complying with Stockholm convention banning of Persistent Organic Pollutant
Heptachlor	2001	(O) Obsolete not classified	Complying with Stockholm convention banning of Persistent Organic Pollutant
BHC	2001	(O) Obsolete not classified	Complying with Stockholm convention banning of Persistent Organic Pollutant
Organomercury fungicides	2001	Ia	Complying with Stockholm convention banning of Persistent Organic Pollutant
Toxaphene	2001	(O) Obsolete not classified	Complying with Stockholm convention banning of Persistent Organic Pollutant
Monocrotophos	2006	Ib	Highly hazardous to human health and environment
Methyl parathion	2006	Ia	Highly hazardous to human health and environment
Endosulfan	2012	II	Highly hazardous to fish and environment
Phorate	2015	Ia	Highly hazardous to human health and environment
Carbofuran	2019	Ib	Highly hazardous to human health and environment
Dichlorvos	2019	Ib	Minimize suicide due to poisoning
Triazophos	2019	Ib	Highly hazardous to human health and environment
Carbaryl	2019	II	Highly hazardous to human health and environment
Benomyl	2019	U	Highly hazardous to human health and environment
Carbosulfan	2019	II	Highly hazardous to human health and environment
Dicofol	2019	II	Complying with Stockholm convention banning of Persistent Organic Pollutant
Aluminum phosphide 3 g tablet	2019	FM (Fumigant not classified)	To minimize suicide cases

In 2019, the Pesticide Registration Board banned eight pesticides including the high concentration (56%) 3 g tablet form of aluminum phosphide as well as several pesticides important for suicide in South Asia (carbofuran, carbosulfan, dichlorvos, and triazophos) (Table 2). Import and production was banned from August 4th, 2019. During the following two years, stockpiles in the country (Table 3) can be sold to farmers; after the two-year phase out period, all remaining stockpiles will need to be deposited in a government-built warehouse.

**Table 3:** Reported Nepalese stockpiles of recently banned pesticides (December 2019)

1	Carbofuran	64,000 kg
2	Aluminum phosphide tablet 3 g	32,500 kg
3	Dichlorvos	44,000 Lt
4	Triazophos	1200 Lt
5	Benomyl	0
6	Carbaryl	0

### Suicides

**Fig. 2:**
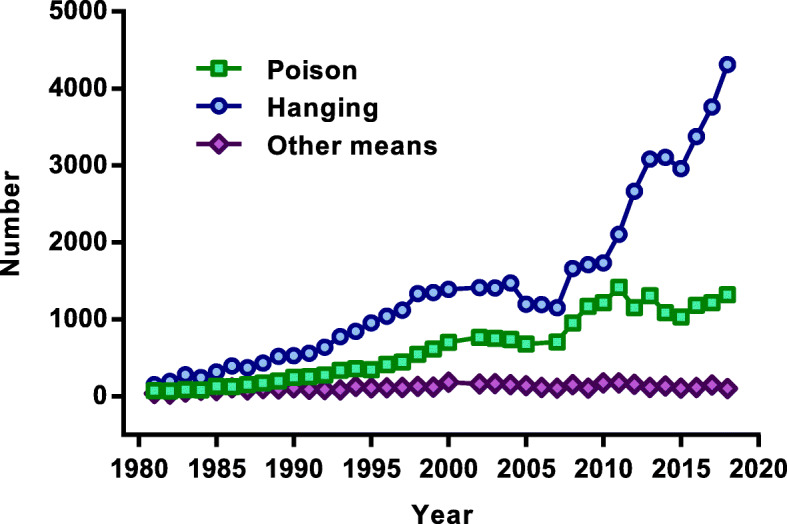
Suicide numbers in Nepal 1980–2019. (Source: Nepal police, data available from the authors upon request)

Suicidal deaths reported to the police have been increasing steadily in the past years (Fig. 2). In 2018–2019, 5754 deaths were reported, compared to 5317 in 2017–2018 and 5124 in 2016–2017 [25]. The calculated annual crude suicide rate in 2018–2019 was 20.7/100,000. The number of suicides in 1980 was recorded as 247. The rate of suicide increased from 1.64/100,000 in 1980 to 20.72/100,000 in 2018–19 (1163% increase).

According to the police data, the most common method of suicide was hanging (4312, 74.1%) in 2018–19. Suicide by poisoning (*n* = 1320) accounted for 22.9% of suicides in the same period [25]. Between 1980 and 2019, hanging and poisoning accounted for 51,749 (66.1%) and 22,153 (28.3%) of all suicide deaths respectively. Hanging accounted for the majority of the increase in suicide rate, increasing from 155 in 1980 (1.03/100,000) to 4312 in 2018–19 (15.35/100,000) (1390% increase, Fig. 2). The rate of increase in poisoning suicides was less, from 55 in 1980 (0.37/100,000) to 1320 in 2018–19 (4.7/100,000) (1170% increase in rate), with most of the increase occurring in 1995–2003, and in 2008–12. Poisoning suicide numbers have been stable over the last 8 years, while hanging has increased greatly.

### Pesticides involved in suicides

**Fig. 3:**
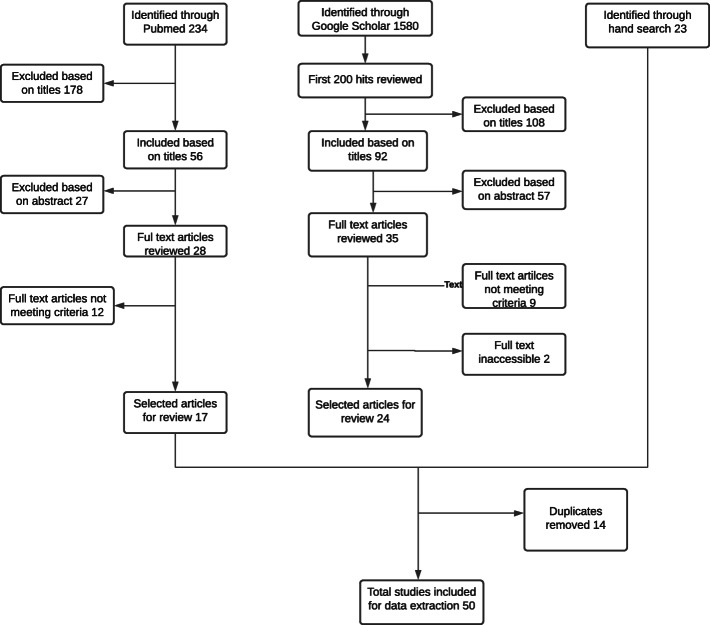
PRISMA Flow diagram

Our search identified 50 relevant publications ([17, 26–74]) (Fig. 3 and Table 4). Studies were published between 1990 and 2020. Most were hospital-based studies published in Nepalese academic journals. The number of poisoned patients in each publication ranged from 37 [26] to 2621 [37].

**Table 4:** Pesticides identified in case series of pesticide self-poisoning in Nepal 1980–2019

#	Year [ref]	Location	Pesticide cases /out of total number of patients	Pesticide classes	Compounds identified	Fatalities with responsible compound (where identified)
1	1995–1996 [26]	Dharan	37/37	All OP	23 Methyl parathion	Not identified
2	2000 [29]	Kathmandu Pokhara Dharan	256/256	133 OP	80 Methyl parathion 53 Dichlorvos 29 Zinc phosphide 18 Aluminum phosphide	42 (16.2%) cases.
3	2003–5 [38]	Kathmandu	47/47	47 OP	32 Methyl parathion	3 fatalities
4	2003–2006 [32]	Dhulikhel	59/59	All OP	15 Dichlorvos 13 Methyl parathion	
5	2003–6 [33]	Pokhara	56/98	32 (31.68%) Rodenticides 15 (14.85%) OP insecticides	Not identified	5/32 Rodenticides 4/15 OP insecticides
6	2004 [34]	Kathmandu	154/154	65 OP 38 Drugs 10 Zinc phosphide 6 Aluminum phosphide 2 Carbamate	31 Dichlorvos 27 Methyl parathion	4/7 Methyl parathion
7	2004–5 [72]	Kathmandu	50/50	All OP poisoning	34/50 Methyl parathion 13/50 Dichlorvos	7 (14%)
8	2005 [35]	Kathmandu	74/99	63 Insecticides 11 Rodenticides	21 Methyl parathion 16 Dichlorvos 5 Aluminium phosphide	2/21 Methyl parathion 1/16 Dichlorvos
9	2005–2008 [36]	Dharan	73/122	55 (45.1%) OP 10 (8.2%) Organochlorine insecticides 4 Cypermethrin 3 Zinc phosphide 1 Carbamate	Not identified	12.6%
10	2005–2010 [37]	Dharan	1661/2621	1101 (67%) OP 321 Organochlorine insecticides 182 (10.96%) Rodenticide	Not identified	168 (6.41%) fatal 156 OP 6 Zinc phosphide
11	2005–11 [74]	Kathmandu	190/354	150 OP 40 Rodenticides 71 Medicine	61 Methyl parathion	19 OP 1 Rodenticide
12	2006–2010 [39]	Pokhara	25/94	6 OP Carbamates 3 Rodenticide	6 Methyl parathion	1 Methyl parathion
13	2006–2012 [40]	Pokhara	34/187	30 OP 4 Carbamates	9 Methyl parathion 5 Dichlorvos 1 Malathion 3 Propoxur (baygon)	1/9 Methyl parathion 1/3 Propoxur
14	2007 [42]	Dhulikhel	54/54	40 OP 3 Zinc phosphide	21 Methyl parathion 16 Dichlorvos	5,5% fatal
15	2007 [43]	Chitwan	182/921	Snake bite 366 (39.7%) Insecticide 182 (19.8%)	84 OP 58 Zinc phosphide 21 Cypermethrin 6 Aluminum phosphide	Not identified
16	2007–2008 [44]	Kathmandu	41/148	29 OP 12 Zinc phosphide	12 Zinc phosphide	
17	2008–2011 [45]	Kathmandu	11/35	6 OP 1 Zinc phosphide 1 Organochlorine	Not identified	Not identified
18	2008 [71]	Pokhara	65/65	All OP	42 Methyl parathion 11 Baygone spray 6 Dichlorvos 3 Malathion	Not identified
19	2008–2011 [46]	Pokhara	96/160	67 OP 23 Rodenticide 6 Cypermethrin	Not identified	8 OP 1 rodenticide 2 cypermethrin
20	2010 [48]	Chitwan	88/178	19 OP 3 Organochlorine 5 Cypermethrin 6 Phosphides	5 Cypermethrin 6 Phosphide 3 Endosulfan	3/19 OP 2 Endosulfan 1 Phosphide
21	2010–11 [73]	Birgunj	171/171	all OP	89 Methyl parathion 22 Malathion 30 Dichlorvos 12 Baygon spray 18 Unknown	18.71% mortality
22	2010–2011 [66]	Dharan	32/149	30 OP 2 Aluminum phosphide	2 Aluminum phosphide	All (study of postmortem cases)
23	2011–12 [50]	Kavre	91/137	56 OP	17 Aluminum phosphide 18 Methyl parathion / Dichlorvos	4/137 Unknown
24	2012–13 [63]	Dharan	763/1399	398 OP 16 Fertiliser	332 OP 175 Phosphide 66 OP in combination 190 Organochlorine	5,1%
25	2013–2015 [51]	Rupandehi	72/107	21 (19.63%) OP 27 (25.23%) Cypermethrin 9 Rodenticide	27 Cypermethrin 9 Dichlorvos	5/107 fatal
26	2013–2017 [52]	Kathmandu	87/144	75 OP 11 Drugs 11 Rodenticide 1 Fungicide	16 Dichlorvos 14 Methyl parathion 13 Cypermethrin 13 Chlorpyrifos + Cypermethrin 6 Chlorpyrifos 5 Dimethoate	7 (4.9%) fatal 3/16 Dichlorvos 1 Aluminum phosphide
27	2014 [53]	Kathmandu	110/84	64 OP 20 Zinc sulphide	Not identified	4 (6.25%)
28	2014–2016 [55]	Lumbini	38/65	21 OP 17 Zinc phosphide	17/38 Zinc phosphide	
29	2014–2017 [56]	Lumbini	87/138	50 OP 23 Rodenticide 2 Fungicide	Not identified	1/50 OP
30	2015 [57]	Kathmandu	84/84	All OP	48 Dichlorvos 20 Methyl parathion 10 Cypermethrin + Chlorpyrifos 4 Triazophos + Deltamethrin	8 fatal
31	2015 [62]	Dharan	35/57	20 OP (24.6%) 15 Zinc phosphide (14%)	Not identified	8.7% mortality
32	2015 [58]	Chitwan	439/439	263 (59.9%) Insecticides 91 (20.8%) Rodenticide 15 (3.4%) Herbicide 5 (1.1%) Fungicide	174 (39.6%) OP 154 (35.1%) Pyrethroid 95 (21.6%) Zinc and Aluminum phosphide 4 Dinitrophenol derivative 2 Carbamate 2 Organochlorine	16 (3.8%)
33	2015–2016 [64]	Manipal	78/78	78 OP	Methyl parathion 24 (30.76%) Dichlorvos 18 (23.07%) Cypermethrin 3 (3.84%) Chlorpyrifos + Cypermethrin 8 (10.25)	5.12%
34	2015–2017 [59]	Pokhara	88/156	45 (28.8%) OP 43 (27.5%) Rodenticide	Not identified	Not identified
35	2016–2018 [60]	Biratnagar	58/85	37 OP 12 Organochlorine 9 Zinc/al phosphide	Not identified	5 OP 10 non-OP
36	2016–2018 [61]	Kathmandu	210/210	All OP poisonings	Methyl parathion 65%	7.62%

All the papers reported Organophosphorus (OP) insecticides to be responsible for most poisonings, ranging from 39.6% [58] to 65.0% of all poisonings [30], but most did not report the exact pesticides used. The second most common poisoning was with aluminium or zinc phosphide (10.7 to 26.1%). Few cases of poisoning and very few fatal cases in these papers were due to non-pesticide poisons. From 87 to 97% of poisoning patients in each publication was due to intentional poisoning. Mortality varied between 3 and 18.7%.

Among the studies that determined the OP pesticide used, methyl parathion and dichlorvos were the most common in all studies reviewed (Table 4). Depending on the study, methyl parathion (Metacid) accounted for 12.9% (2005–2011) [74], 17.5% (2004) [68], 52% (2012) [73] and 65–68% (2003–2005 and 2016–2018) [38, 61] of all OP insecticide poisonings (Table 4). Dichlorvos (Nuvan) was the compound used in 17 to 24% of OP poisoning. Malathion was the third most commonly used OP agent for poisoning [73]. Monocrotophos was not reported in any publication.

## Discussion

There is no systematic system for suicide surveillance in Nepal [20]. Suicide and attempted suicide cases need to be reported to the police but due to the stigma and perceived (not actual) illegality of the act, many cases are believed to not be reported, leading to under-reporting of national data [16, 20]. In addition, there is misclassification as well as gaps in hospital record-keeping. This is a common situation across South Asia in countries with an uncertain legal status for suicide [75]. A small anomaly in Nepal is that group suicides are recorded by the police as a single suicide, further reducing numbers.

The WHO presents a wide range of competing estimates for suicide in Nepal, both considerably higher and lower than the official statistics. In 2014, the WHO modelled a 2012 predicted suicide rate, ranking Nepal 7th in the world at 24.9 per 100,000, the 3d highest for women (20 per 100,000) and 17th for men (30.1 per 100,000) [13]. In contrast, the WHO Global Health Estimates for 2019 reports 2544 suicides in Nepal (markedly lower than the 5819 reported by the police), of which 1176 (8.0/100,000) occurred in females and 1368 (11.4) occurred in males. This equates to an overall suicide rate of 9.6/1000,000, lower than the mean global rate of 10.5/100,000 population [13]. The discrepancy in numbers shows the importance of developing reliable national data on suicides and pesticide suicides.

Nepal’s hospital staff have difficulties identifying the compounds responsible for poisoning for patients who present to hospitals. In most cases, when the poisoning agent is recorded in hospital records, the patient or their family brought the containers to the hospital. When the compound used is not known, the atropine challenge test is used to determine if the patient ingested an OP chemical [37]. For the non-OP pesticides, the diagnosis is usually based on the container or information from the patient or family. In cases of OP poisoning determined by the atropine challenge test, the exact compound is usually unknown. If there is no container of the ingested compound and the atropine challenge is negative, the name of the agent is not indicated in the medical notes, making it difficult to identify the responsible agent.

We found that the number of suicides in Nepal have increased markedly since 1980, mostly due to a great increase in suicides from hanging. Pesticide suicides have made up about ¼ of suicides during this time but have not increased in number to the same extent. The most important pesticides responsible for pesticide poisonings and deaths in the literature has been OP pesticides. Most frequently used was methyl parathion (Metacid); although banned in 2006, it still appears as widely used poisoning agent, suggesting that the pesticide may have come across the border from India. However, very few patients bring the pesticide bottles with them to hospital and the pesticide is named from memory. Since Metacid is a widely used pesticide, it is possible that the term Metacid is used generally for any pesticides as occurred in Sri Lanka with Folidol (another brand name for methyl-parathion) and then Tamaron (methamidophos). This habit may have led to an overestimation of the importance of methyl parathion as poisoning agent. Our recent data from forensic science laboratories which identify the actual compounds ingested in fatal poisoning suggest that methyl-parathion poisoning is not a significant problem in Nepal anymore (Ghimire et al., submitted).

The other important pesticides for fatal self-poisoning were dichlorvos and aluminum phosphide, which were either banned completely (dichlorvos) or high concentration formulations banned (aluminum phosphide) in 2019.

Other pesticides have become increasingly frequently identified as involved in pesticide poisoning, including cypermethrin, chlorpyrifos, and malathion. Since the historically most important pesticides for suicide (methyl-parathion, dichlorvos and aluminum phosphide tablets) are now banned in Nepal, it will be important to observe for changes in the Nepal’s suicide rate. In this context, implementation of the regulations and elimination of the illegal sales become very important, as well as providing farmers with advice on substitution of banned pesticides. Based on data from elsewhere in Asia [76], these HHPs bans should result in a marked fall in pesticide suicides over the next 2–5 years.

Nepal has been successful at reducing the number of HHPs used in its agriculture, despite the increase in use of pesticides. This likely accounts for the very low number of occupational poisoning cases we have found to be admitted in hospital in Nepal (Ghimire, submitted). There are now only two WHO class Ia (bromadiolon) and Ib (Zinc Phosphide) pesticides left in use in Nepal. The necessity of their use should be reviewed by the PQPMC and removed if they cannot be used safely. As the recent bans are enacted and stockpiles run out, it will be important to see which WHO hazard class II insecticides become problematic for suicide, requiring an analysis of their need in agriculture.

### Limitations

We used police data for suicide deaths. Due to stigma and fear of perceived negative consequences, this likely underrepresents the actual numbers of suicides. To identify the agent used for poisoning we reviewed the literature to identify the agents. This is less reliable than accurate hospital based collection of data on the poisoning agent and ideally identification of the actual compound through forensic toxicology laboratory analysis. In addition, there is misclassification as well as gaps in hospital record-keeping.

## Conclusion

In the last twenty years (2001–2019), Nepal has banned many HHPs, including the ones that were commonly used for pesticide self-poisoning. During this time, the incidence of hanging has risen markedly with a much smaller increase in pesticide suicides, which might be related to the regulation. Research is now needed on the effect of the 2019 ban of eight pesticides, many of which have been key for pesticide suicides over the last 15 years.

## Data Availability

Data and materials are available from Leah Utyasheva upon request.

## Abbreviations

a.i: active ingredient
AKC: Agriculture Knowledge Centre
HHPs: Highly Hazardous Pesticides
HMIS: Health Management Information System
LMIC: Low-and Middle-Income Country
MoALD: Ministry of Agriculture and Livestock Development
OP pesticide: Organophosphorus pesticide
PADD: Provincial Agriculture Development Directorate
POP: Persistent Organic Pollutants
PQPMC: Plant Quarantine and Pesticide Management Centre
SDGs: Sustainable Development Goals
WHO: World Health Organization
